# Generation of an Fsp1 (fibroblast‐specific protein 1)‐Flpo transgenic mouse strain

**DOI:** 10.1002/dvg.23359

**Published:** 2020-03-19

**Authors:** Victoire Cardot‐Ruffino, Véronique Chauvet, Cassandre Caligaris, Adrien Bertrand‐Chapel, Nicolas Chuvin, Roxane M. Pommier, Ulrich Valcourt, David Vincent, Sylvie Martel, Sophie Aires, Bastien Kaniewski, Pierre Dubus, Philippe Cassier, Stéphanie Sentis, Laurent Bartholin

**Affiliations:** ^1^ INSERM U1052, Centre de Recherche en Cancérologie de Lyon Lyon France; ^2^ CNRS UMR5286, Centre de Recherche en Cancérologie de Lyon Lyon France; ^3^ Université de Lyon Lyon France; ^4^ Université Lyon 1 Lyon France; ^5^ Centre Léon Bérard Lyon France; ^6^ Beatson Institute for Cancer Research Glasgow UK; ^7^ INSERM, Univ Bordeaux UMR1053 Bordeaux Research in Translational Oncology Bordeaux France; ^8^ CHU de Bordeaux Bordeaux France; ^9^ Departement d'Oncologie Médicale Centre Léon Bérard Lyon France; ^10^ Present address: Clinical Research Division, Fred Hutchinson Cancer Research Center Seattle Washington USA; ^11^ Present address: Laboratoire de Biologie Tissulaire et Ingénierie Thérapeutique, UMR 5305 CNRS ‐ Université Lyon 1, Institut de Biologie et Chimie des Protéines Lyon France; ^12^ Present address: Dana Farber Cancer Institute Boston Massachusetts USA

**Keywords:** fibroblast, Fsp1, Flpo, mice, recombinase, transgenic

## Abstract

Recombination systems represent a major breakthrough in the field of genetic model engineering. The Flp recombinases (Flp, Flpe, and Flpo) bind and cleave DNA Frt sites. We created a transgenic mouse strain ([Fsp1‐Flpo]) expressing the Flpo recombinase in fibroblasts. This strain was obtained by random insertion inside mouse zygotes after pronuclear injection. Flpo expression was placed under the control of the promoter of *Fsp1* (fibroblast‐specific protein 1) gene, whose expression starts after gastrulation at Day 8.5 in cells of mesenchymal origin. We verified the correct expression and function of the Flpo enzyme by several ex vivo and in vivo approaches. The [Fsp1‐Flpo] strain represents a genuine tool to further target the recombination of transgenes with Frt sites specifically in cells of mesenchymal origin or with a fibroblastic phenotype.

## 1 INTRODUCTION

The Flp/Frt recombination system was discovered in *Saccharomyces cerevisiae* (Broach, 1982). At the molecular level, the Flp recombinase enzyme binds and cleaves DNA Frt sites. Active at a temperature range of 25–30°C, the Flp recombinase enzyme was successfully transfer into drosophila to generate conditional genetically‐modified flies (Golic & Lindquist, 1989). The Flpe recombinase is an optimized Flp, developed using molecularly advanced tools to improve its efficacy at higher temperatures (37–38°C; Buchholz, Angrand, & Stewart, 1998). In 2007, a codon‐optimized version of Flpe recombinase called Flpo recombinase was generated for optimal efficacy in vitro in mammalian cells (Raymond & Soriano, 2007), and was further demonstrated to be highly efficient in vivo in mice (Kranz et al., 2010; Raymond & Soriano, 2010; Y. Wu, Wang, Sun, LeRoith, & Yakar, 2009). Flpe and Flpo (Flpe/o) recombinases were first used in embryonic stem (ES) cells to excise Frt‐flanked antibiotic‐resistant cassettes (neomycin, puromycin…) in order to generate mice that do not express the antibiotic resistance gene used for the selection of ES cells (Schaft, Ashery‐Padan, van der Hoeven, Gruss, & Stewart, 2001; Testa et al., 2004). The development of mouse strains ubiquitously expressing the Flpe/o (“Flip deleter” strains) enabled scientists to conduct murine in vivo recombination experiments (hACTB:Flp, (Dymecki, 1996); CAG:FLPe, (Rodriguez et al., 2000); ROSA26‐Flpo (Raymond & Soriano, 2010); PGK‐Flpo, (Y. Wu et al., 2009); pCAGGS‐Flpo, (Kranz et al., 2010)). Moreover, mouse strains expressing Flpe/o in specific organs or compartments were created targeting specific organs, including the brain/neurons (rPOMC (Vooijs, van der Valk, te Riele, & Berns, 1998), Phox2b (Hirsch, d'Autreaux, Dymecki, Brunet, & Goridis, 2013), Bhlhb5 (Cai et al., 2016), Wnt1 (Farago, Awatramani, & Dymecki, 2006), Atoh1 (van der Heijden & Zoghbi, 2018), or Nkx2.1 (He et al., 2016)), and pancreas (Pdx1 (Schonhuber et al., 2014), (J. Wu et al., 2017)). In parallel, several FRT conditional mouse strains were generated for cell lineage tracking (using GFP (Jensen et al., 2008; Yamamoto et al., 2009), mCherry (Niederkofler et al., 2016), tdTomato (Plummer et al., 2015) fluorescent proteins, and alkaline phosphatase (R26:FRAP) enzyme (Awatramani, Soriano, Mai, & Dymecki, 2001)), as well as gene knockout (e.g., Trp53^FRT^ [Lee et al., 2012]) and gene knock‐in procedures (e.g., Kras^FSF‐G12D^ [Young, Crowley, & Jacks, 2011]). The Flpo^ER^ system was then established to control temporal expression of Flpo, upon tamoxifen injection into mice (Goodrich, Talhouk, Zhang, & Goodrich, 2018; Lao, Raju, Bai, & Joyner, 2012). Recently, a light‐sensitive Flpo recombinase was developed as a nontoxic and noninvasive alternative to tamoxifen (Flpo recombinase active heterodimers can form after LED light illumination of a specific zone [Jung et al., 2019]). Despite these advances, the development of organ‐ or cell‐specific “Flp” mice remains limited, hindering the possibility of taking advantage of the Flp/Frt recombination system in vivo.

Considering the crucial role of fibroblasts in the microenvironment, we created in the present study an original mouse model expressing the Flpo recombinase in the fibroblastic compartment ([Fsp1‐Flpo] mouse strain). We chose the Flp/Frt approach with the final goal in the future of combining this system with the Cre/lox system in dual recombinase systems (DRS), which consist in combining two recombinase systems within the same mouse (e.g., Cre/Lox and Flp/Frt).

## 2 RESULTS AND DISCUSSION

**Figure 1:**
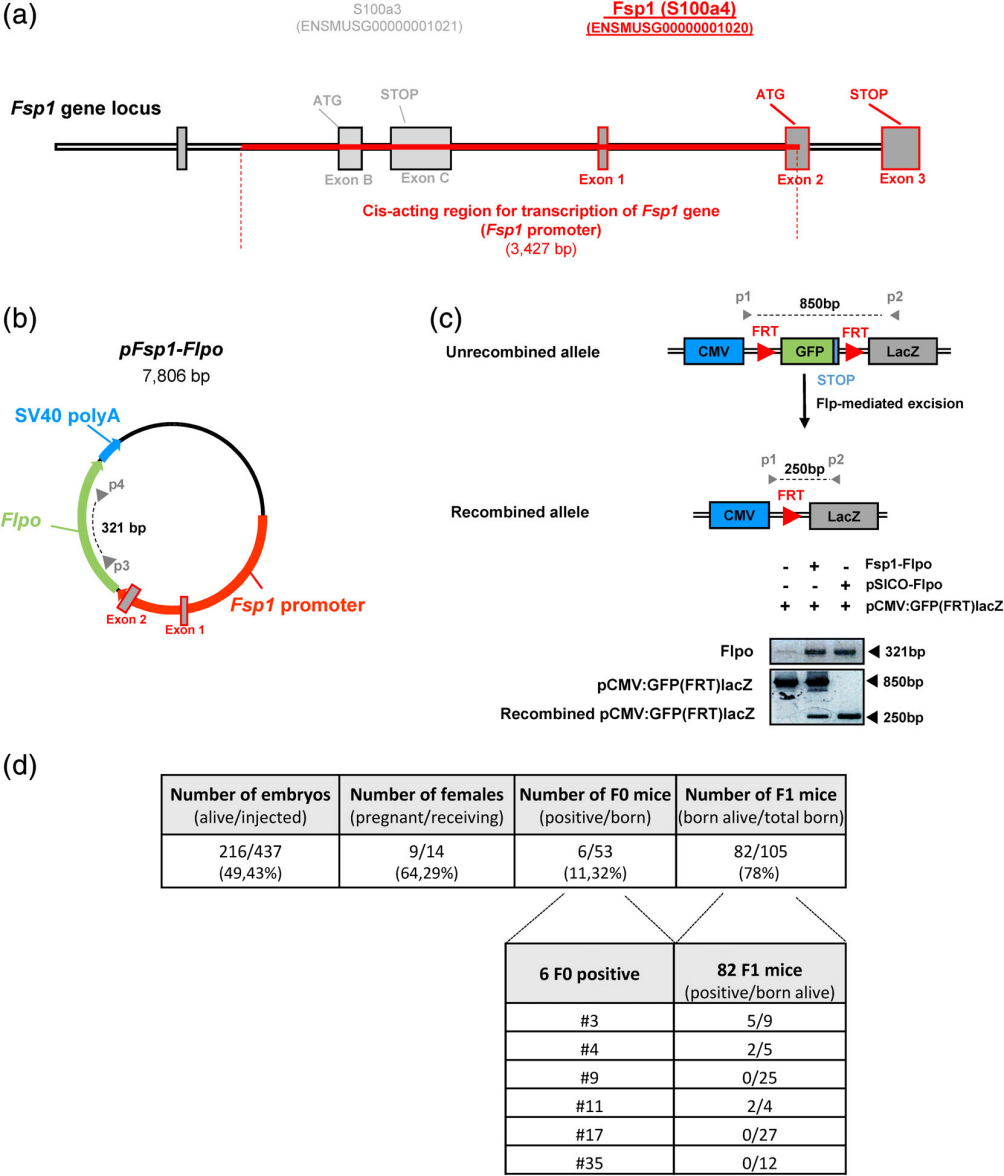
Generation of the [Fsp1‐Flpo] mouse strain. (a) *Fsp1* gene locus in mouse. (b) Circular map of the pFsp1‐Flpo plasmid: *Fsp1* promoter was cloned into the Flpo transgenesis plasmid. Primers used for the PCR in Figure 1c and RT‐PCR in Figures 2a, b, d are represented by gray arrowheads (p3‐4). (c) Schematic diagram of the Flpo‐mediated excision (top panel) and PCR on DNA (bottom panel) after transient co‐transfection of pCMV:GFP(FRT)lacZ reporter along with pFsp1‐Flpo or pSICO‐Flpo control vector in HaCaT cells. (d) Table recapitulating the number of mice generated/obtained at each step of the transgenesis strategy leading to the generation of [Fsp1‐Flpo] mice

The *Fsp1* promoter consists in a ~3 kbp genomic DNA fragment that drives gene expression in fibroblasts (Bhowmick et al., 2004; Iwano et al., 2002; Okada et al., 1998; Strutz et al., 1995). The *Flpo* transgene is a mouse codon‐optimized Flp (Flpo) site‐specific recombinase (SSR), which recombines DNA Frt‐sites (Raymond & Soriano, 2007). A *Fsp1* promoter fragment (Exon_1/Intron_1/Partial_Exon_2; ENSMUSG00000001020) corresponding to the region previously described to drive the expression of Fsp1 in fibroblasts (Figure 1a) was amplified by PCR from the CH29‐508C7 CHORI BAC clone. After subcloning in a pBluescript plasmid (pBS), this promoter fragment was inserted upstream of Flpo creating the pFsp1‐Flpo plasmid (size 7,806 bp) (Figure 1b). This vector was fully verified by DNA sequencing (Figure S1). The pFsp1‐Flpo plasmid was further functionally validated in vitro by transient co‐transfection in HaCaT cells (expressing Fsp1, data not shown) of a reporter with a Flp excisable GFP cassette **(**Figure 1c**)** along with pFsp1‐Flpo vector or a control vector pSICO‐Flpo constitutively expressing the Flpo recombinase (under the control of the pGK promoter; Young & Jacks, 2010). PCR experiments performed on genomic DNA prepared from transfected cells revealed the recombined pCMV:GFP(FRT)lacZ transgene only when Flpo was present (Figure 1c). Of note, recombination efficacy was lower with the pFsp1‐Flpo plasmid than the pSICO‐Flpo plasmid, resulting likely from differences in promoter activities and/or transfection efficiencies. The validated pFsp1‐Flpo vector was injected into 437 oocytes (FVB × B6 × DBA2 mixed background) and 216 embryos survived injection **(**Figure 1d**)**. These embryos were further transferred into 14 OF1 foster females for gestation and we obtained 9 pregnant females giving birth to 53 progenies (F0 generation), 6 of which were positive for the transgene. To verify germinal transmission of the transgene, we bred these 6 F0 individuals with C57BL6/J mice and obtained 105 progenies (82 born alive) (F1 generation), of which 9 heterozygous Fsp1 were identified from 3 different F0 ([Fsp1‐Flpo]‐F0#3, ‐F0#4, ‐F0#11) **(**Figure 1d**)**. Following another backcross in a C57BL6/J background, [Fsp1‐Flpo]‐F0#4 and its offspring were discarded as the transgene appeared to be inserted on a sexual chromosome.

**Figure 2:**
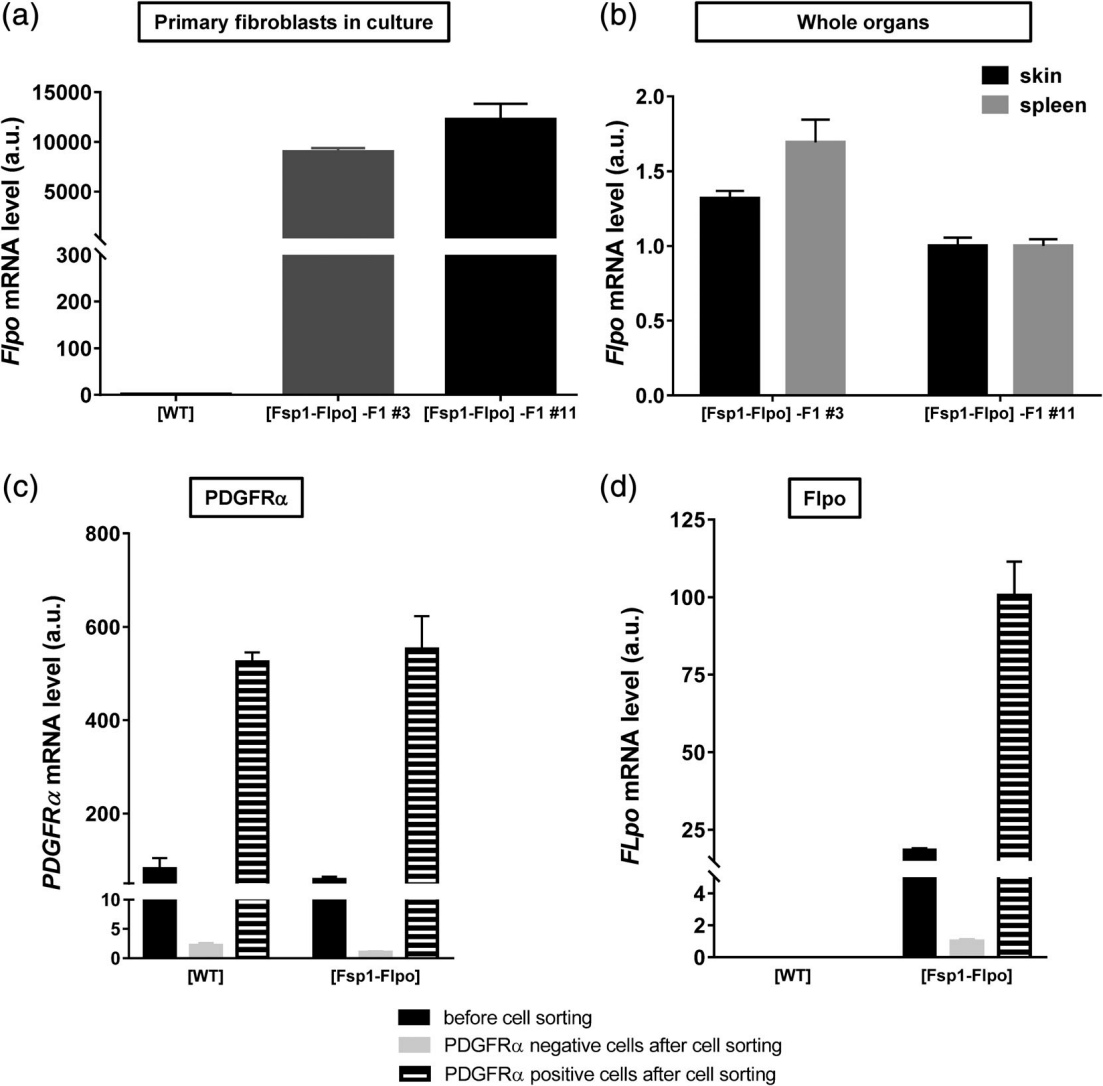
Expression of the *Fsp1‐Flpo* transgene in the [Fsp1‐Flpo] mouse strain. Quantification of *Flpo* mRNA by RT‐qPCR performed on total mRNA prepared from primary fibroblasts in culture (a) and whole organ tissues (skin and spleen) (b) from one wild‐type mouse ([WT]) and two F1 heterozygous founders ([Fsp1‐Flpo]‐F1#3 and [Fsp1‐Flpo]‐F1#11). Quantification of *PDGFRα* (c) and *Flpo* (d) mRNA by RT‐qPCR on total RNA prepared from [WT] and [Fsp1‐Flpo] cells present in back skin before and after cell sorting of PDGRα‐positive cells (PDGFRα‐positive cells represent cells binding to the anti‐PDGFRα antibody and PDGFRα‐negative cells represent cells with no binding to anti‐PDGFRα antibody)

To detect *Flpo* transcripts, we performed RT‐qPCR on total RNA prepared from [WT] and [Fsp1‐Flpo]‐F1#3 and [Fsp1‐Flpo]‐F1#11 cultured ear skin primary fibroblasts, and, as expected, we detected the expression of *Flpo* mRNA exclusively in fibroblasts bearing the *Flpo* transgene (Figure 2a). *Flpo* transcripts were next quantified in [Fsp1‐Flpo]‐F1#3 and [Fsp1‐Flpo]‐F1#11 RNA extracts from spleen and skin, two organs known to express high levels of *Fsp1* (https://www.proteinatlas.org/ENSG00000196154-S100A4). We observed a slightly higher expression in [Fsp1‐Flpo]‐F1#3 (Figure 2b), and this latter founder was further selected (along with its offspring) for subsequent studies and these were all designated as “[Fsp1‐Flpo]” hereafter. In order to validate the expression of the *Flpo* transgene specifically in the fibroblasts, we dilacerated and homogenized back skin samples from [WT] and [Fsp1‐Flpo] mice to obtain a suspension of individual cells. We then isolated by cell sorting the skin fibroblasts using an antibody directed against PDGFRα (platelet‐derived growth factor receptor α) a pan‐fibroblast marker (Sharon, Alon, Glanz, Servais, & Erez, 2013). In the skin of both genotypes, RT‐PCR highlighted the presence of PDGFRα‐positive cells, PDGFRα being almost undetectable in sorted PDGFRα‐negative cells (cells remaining after the sorting of PDGFRα cells) (Figure 2c). As expected, *Flpo* transcripts were detected only in cells from [Fsp1‐Flpo] mice (no expression in cells from [WT] mice), a >4‐fold enrichment being observed in PDGFRα‐positive cells compared to cells before cell sorting (Figure 2d).

**Figure 3:**
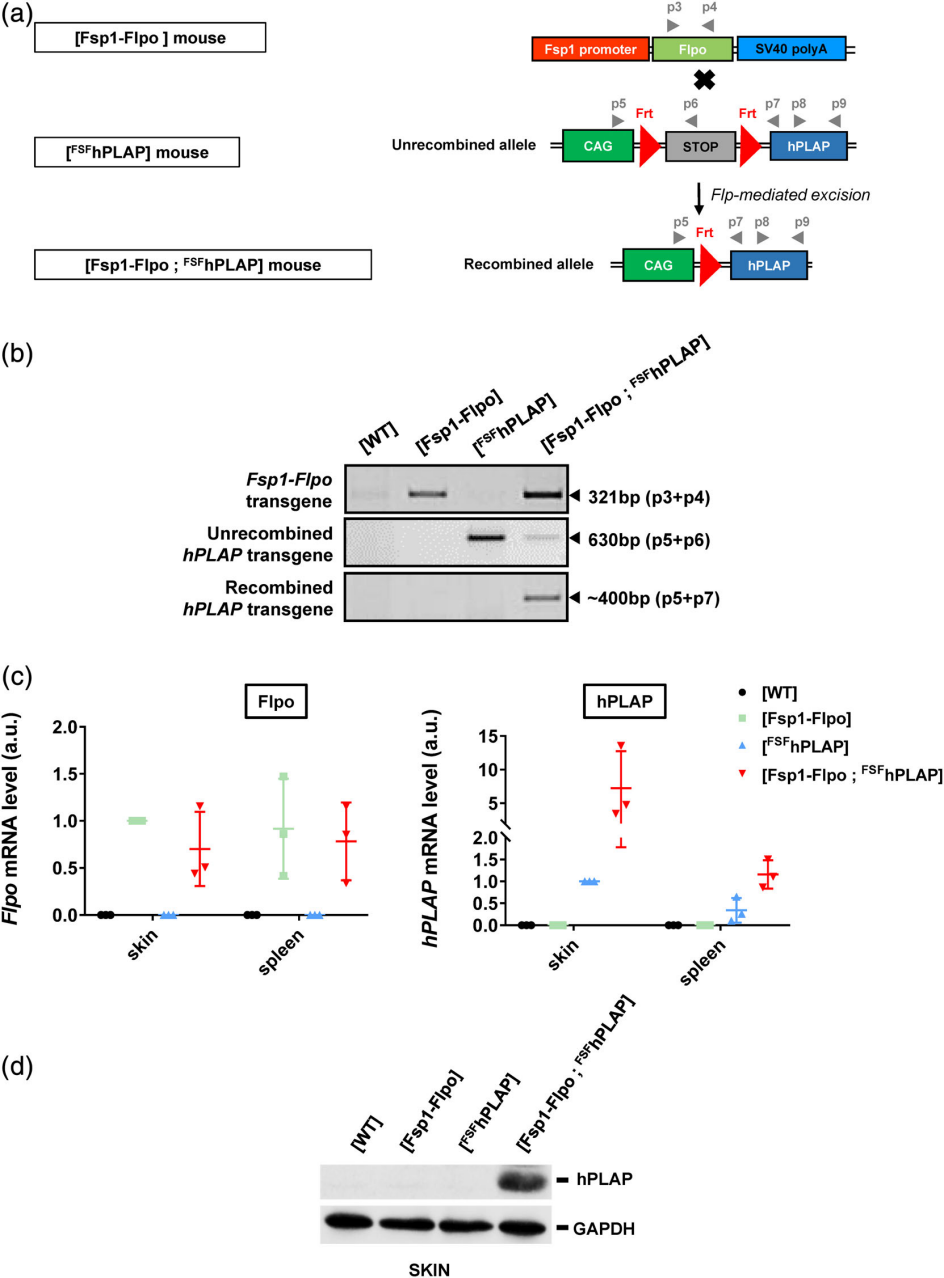
In vivo functional validation of the [Fsp1‐Flpo] mouse strain. (a) Breeding strategy ([Fsp1‐Flpo] x [^FSF^hPLAP]) to generate [Fsp1‐Flpo; ^FSF^hPLAP] individuals. Primers used for DNA genotyping in Panel 3b and RT‐PCR in Panel 3c are represented by gray arrowheads (p3‐9). (b) PCR on genomic DNA prepared from ear skin samples of indicated genotypes to detect the *Fsp1‐Flpo*, unrecombined *hPLAP* and recombined *hPLAP* alleles. (c) Quantification of *Flpo*, *and hPLAP* mRNA by RT‐qPCR on total RNA prepared from [WT], [Fsp1‐Flpo], [^FSF^hPLAP], and [Fsp1‐Flpo; ^FSF^hPLAP] whole skin and spleen extracts. (d) Western blot analysis of hPLAP, GAPDH on whole protein extracts prepared from skin samples of indicated genotypes

In order to detect in vivo *Fsp1‐Flpo* transgene expression, we used the [^FSF^hPLAP] reporter mouse strain bearing a conditional *hPLAP* (human PLacental Alkaline Phosphatase) transgene under the control of a Frt‐Stop‐Frt (FSF) cassette (Awatramani et al., 2001). In cells expressing the Flpo recombinase, the STOP cassette is excised leading to the expression of the *hPLAP* transgene, the activity of which is then detectable by a colorimetric enzymatic reaction. We bred [Fsp1‐Flpo] and [^FSF^hPLAP] mice to generate [Fsp1‐Flpo; ^FSF^hPLAP] individuals with two transgenes (Figure 3a). PCR on genomic DNA showed that the recombined allele was only detectable in the ear skin samples from [Fsp1‐Flpo; ^FSF^hPLAP] mice (Figure 3b). RT‐qPCR (Figure 3c**)** were performed on total RNA prepared from skin and spleen samples from [WT], [Fsp1‐Flpo], [^FSF^hPLAP], or [Fsp1‐Flpo; ^FSF^hPLAP] mice. Flpo mRNA was detected only in organs of [Fsp1‐Flpo] and [Fsp1‐Flpo; ^FSF^hPLAP] mice (Figure 3c left‐hand panel). hPLAP was highly expressed in skin of [Fsp1‐Flpo; ^FSF^hPLAP] mice but was much lower in their spleen (close to the level due to the leakage in [^FSF^hPLAP] mice; Figure 3c right‐hand panel). Western blot analyses performed on whole protein extracts from skin and spleen samples confirmed the presence of the hPLAP protein exclusively in [Fsp1‐Flpo; ^FSF^hPLAP] skin samples (Figure 3d), whereas it was barely or no detectable in spleen (data not shown).

**Figure 4:**
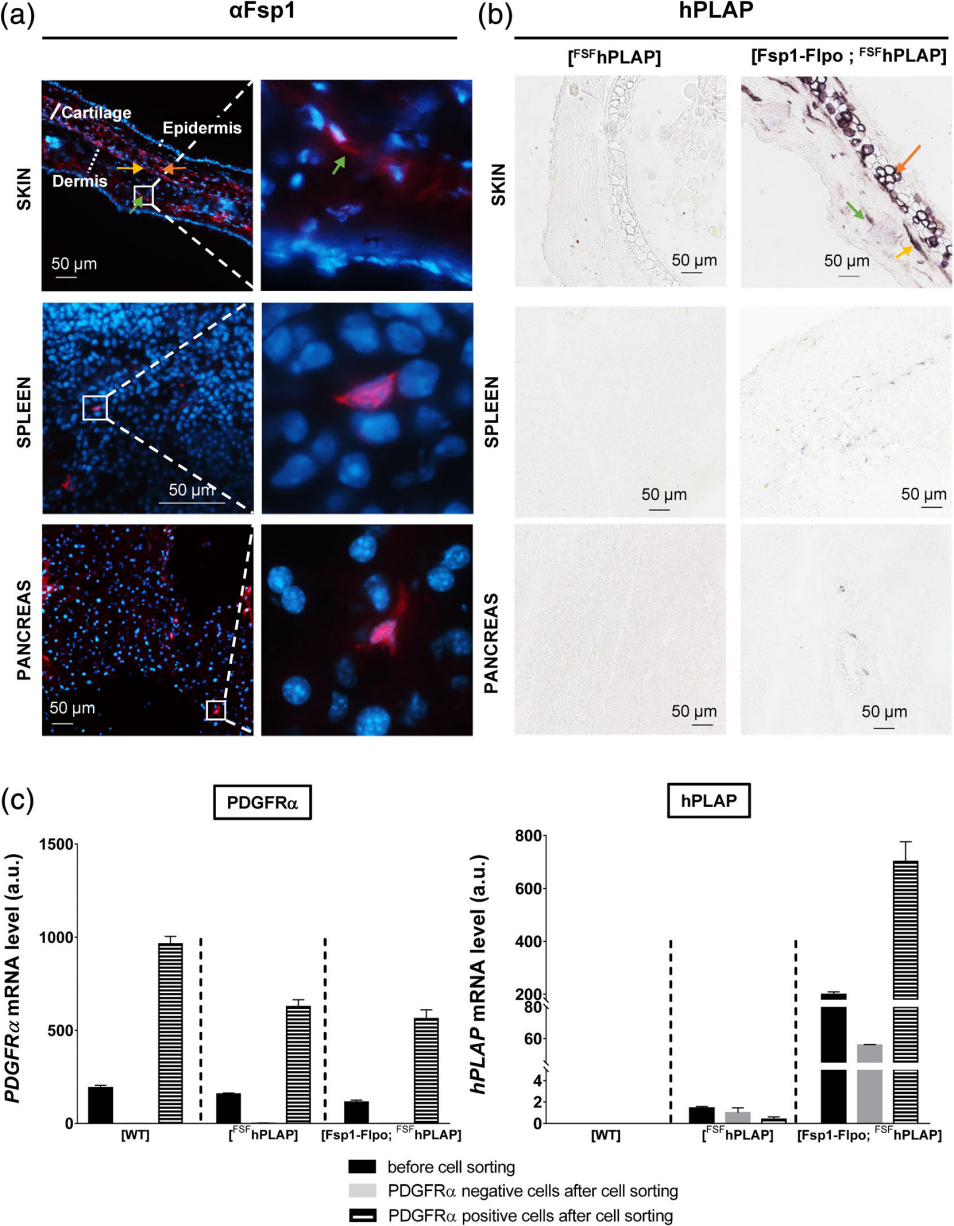
In vivo cellular specificity of the *Flpo* transgene expression. (a) Immunofluorescence detection of Fsp1 protein in normal skin, spleen and pancreas. (b) Enzymatic detection of hPLAP activity in skin, spleen and pancreas from [Fsp1‐Flpo; ^FSF^hPLAP] mice and [^FSF^hPLAP] control mice. (c) Quantification of PDGRα (left‐hand panel) and hPLAP (right‐hand panel) mRNA by RT‐qPCR on total RNA prepared from [WT], [^FSF^hPLAP], and [Fsp1‐Flpo; ^FSF^hPLAP] cells present in back skin before and after cell sorting of PDGRα‐positive cells (PDGFRα‐positive cells represent cells binding to the anti‐PDGFRα antibody and PDGFRα‐negative cells represent cells with no binding to anti‐PDGFRα antibody). Yellow arrow: fibroblasts surrounding chondrocytes; green arrow: dermis fibroblasts; orange arrow: chondrocytes

Next, we first performed immunofluorescence (IF) experiments on skin, spleen, and pancreas tissue sections. We observed that the Fsp1 protein was expressed in dermis fibroblasts (Figure 4a top panels, green arrow), in fibroblasts surrounding the elastic cartilage (Figure 4a top panels, yellow arrow) and in chondrocytes (Figure 4a top panels, orange arrow). This observation corroborates the previously reported expression of Fsp1 in chondrocytes in the elastic cartilage and fibrocartilage (Teng, Kanasaki, Bardeesy, Sugimoto, & Kalluri, 2011). Of note, no Fsp1 protein was detected in epidermis devoid of fibroblasts. The Fsp1 protein was detectable to a lesser extent in some scarce cells in the spleen (Figure 4a middle panels) and pancreas (Figure 4a bottom panels). Regarding the identity of Fsp1‐positive cells in spleen, those are reminiscent of resident fibroblasts as well as of certain populations of leucocytes as previously reported (Boomershine et al., 2009). In the pancreas, Fsp1‐positive cells are reminiscent of resident fibroblasts as well as of histiocytes, axons, neutrophilic granulocytes and mast cells (Nielsen, Mortensen, & Detlefsen, 2017). In situ expression of the Flpo protein was indirectly assed by detecting the enzymatic activity of hPLAP (Figure 4b). In the three organs, the hPLAP staining pattern was quite similar to the Fsp1 protein expression pattern observed by IF (see Figure 4a), demonstrating the specificity of the *Flpo* transgene expression in Fsp1‐positive cells. Hence, in skin, hPLAP activity was restricted to the dermis of [Fsp1‐Flpo; ^FSF^hPLAP] skin (Figure 4b top panel, green arrow), the fibroblasts surrounding the elastic cartilage (Figure 4b top panel, yellow arrow), and chondrocytes (Figure 4b top panel, orange arrow). As expected, in the spleen (Figure 4b middle panel) and pancreas (Figure 4b bottom panel), we observed scarce stained cells likely corresponding to resident fibroblasts in those tissues. Finally, we isolated by cell sorting back skin fibroblasts using the PDGFRα antibody (Figure 4c left‐hand panel). *hPLAP* mRNA was not detected in [WT] mice (Figure 4c right‐hand panel), whereas it was highly expressed in the skin of [Fsp1‐Flpo; ^FSF^hPLAP] mice. *hPLAP* mRNA was barely detectable in [^FSF^hPLAP] skin. The amount of *hPLAP* mRNA was significantly higher after cell sorting in PDGFRα‐positive cells from [Fsp1‐Flpo; ^FSF^hPLAP] mice.

Collectively, these data demonstrate that [Fsp1‐Flpo] mice express a functionally active Flpo recombinase specifically in Fsp1‐positive cells.

To target the expression of Frt‐flanked genes in fibroblasts, we created the *Fsp1‐Flpo* allele encoding the Flpo recombinase under the control of the *Fsp1* promoter. We functionally validated this allele by showing that *Flpo* expression was restricted to Fsp1‐positive cells. We were able to observe in situ the expression of the Fsp1 protein in fibroblasts directly with an anti‐Fsp1 antibody, and Flpo recombinase activity with an in vivo reporter system ([Fsp1‐Flpo; ^FSF^hPLAP]). The *Fsp1* promoter has been successfully used by other groups to generate [Fsp1‐Cre x TβRII^LoxP/LoxP^] mice carrying homozygous inactivation of type II TGFβ receptor in the stromal compartment (Bhowmick et al., 2004; Trimboli et al., 2008). Tissue‐specific Flp drivers and Frt‐flanked transgenes remain rare compared to tissue‐specific Cre drivers and Floxed transgenes. Consequently, the development of DRS (Schonhuber et al., 2014) has been hindered, although they are very effective in allowing the spatiotemporal uncoupling of different transgene expressions in different cell compartments in a single mouse (Meinke, Bohm, Hauber, Pisabarro, & Buchholz, 2016; Schonhuber et al., 2014). The generation of the [Fsp1‐Flpo] mouse strain represents a very valuable tool to target the microenvironment in the context of conditional mutations under the control of the Cre/Lox system targeting epithelial or immune compartments for instance. Uncoupled spatiotemporal regulation of different genetic alterations using DRS should enable scientists to develop models better mimicking the complexity and heterogeneity of human diseases.

## 3 METHODS

### 3.1 Biological material

#### 3.1.1 Cells

**HaCaT** keratinocytes (DKFZ, Heidelberg, 300493) were cultured in complete medium (Dulbecco's Modified Eagle's Medium, supplemented with 0.03% l‐glutamine and containing 10% fetal bovine serum, a mix of 100 U ml^−1^ penicillin and 100 μg ml^−1^ streptomycin sulfate) and propagated at 37°C under 5% (v/v) CO_2_ atmosphere. Primary ear fibroblasts were isolated and cultured as follows: mouse ears were rinsed with 70% ethanol and samples of a few square‐millimeters were harvested. Primary ear fibroblasts were isolated using mechanical dilaceration, followed by enzymatic dissociation (600 μl of a mix of collagenase D (4 mg ml^−1^, COLLD‐RO Roche)/Dispase II (4 mg ml^−1^, Roche) in RPMI medium) at 37°C for 1 hr. The reaction was stopped by adding 5 ml of complete medium (Dulbecco's Modified Eagle's Medium, supplemented with 0.03% l‐glutamine and containing 10% fetal bovine serum, a mix of 100 U ml^−1^ penicillin and 100 μg ml^−1^ streptomycin sulfate) and cells were then incubated at 37°C overnight. The following day, after filtration through a 100 μm pore cell strainer, cells were pelleted and reseeded in complete medium (Dulbecco's Modified Eagle's Medium, supplemented with 0.03% l‐glutamine and containing 10% fetal bovine serum, a mix of 100 U ml^−1^ penicillin and 100 μg ml^−1^ streptomycin sulfate, 1% MEM nonessential amino acids and 50 μM β‐mercaptoethanol). Medium was changed after 48 hr. All cells were propagated at 37°C under 5% (v/v) CO_2_ atmosphere.

*Sorted skin cells* were prepared as follows: mice were sacrificed and shaved, and a piece of skin from the back of the animals was harvested and fat was removed. Five milliliter of digestion medium (RPMI1640, 20% FBS, 1% PS, 1% Hepes, 1% Glutamine) was added to the skin in a petri dish. The skin was dilacerated using scissors and a cutter. Another 5 ml of digestion medium was added to help harvest the mixture. The mixture was then transferred to 50 ml tubes under agitation with a magnetic bar. One milliliter of collagenase type IA (10 mg/ml, C2674‐1G Merck) and 200 μl of DNase1 (10 mg/ml, 11,284,932,001 Merck) were added. The mix was agitated 30–90 min at 37°C. The insoluble debris were eliminated by filtration onto a nylon grid. The digestion product was then centrifuged 5 min at 1,300 rpm and suspended in cell sorting buffer (1X PBS, 1% BSA, 0.5 mM EDTA). Cells were labeled in cell sorting buffer with PE anti‐mouse CD140a/PDGFRα antibody (BioLegend Cat. No. 135905 1:50) and PE Rat IgG2a, κ Isotype Control Antibody (BioLegend Cat. No. 400507 1:50). Cells were sorted using the BD FACS Aria III SORP (BECTON DICKINSON [BD™]).

#### 3.1.2 Mice

The [Fsp1‐Flpo] mouse strain will be available to the research community upon acceptance of the manuscript.

The [^FSF^hPLAP] strain was previously described (Awatramani et al., 2001) and was obtained from Charles River Laboratories ((*Gt(ROSA)26Sor* ^*tm1(ALPP)Dym*^), Stock No: 009086). Mice were housed and bred in the “AniCan” specific pathogen‐free animal facility of the CRCL (Centre de Recherche en Cancérologie de Lyon), France. The experiments were performed in compliance with the animal welfare guidelines of the European Union and with the French legislation (CECCAPP protocol #CLB‐2012‐012 #CLB‐2017‐007 #CLB‐2018‐025).

### 3.2 Cell biology

#### 3.2.1 Cell transfection

At Day 1, 300,000 HaCaT keratinocytes were plated in 12‐well plates. At Day 2, cells in 1 ml of complete medium (Dulbecco's Modified Eagle's Medium, supplemented with 0.03% l‐glutamine and containing 10% fetal bovine serum, a mix of 100 U ml^−1^ penicillin and 100 μg ml^−1^ streptomycin sulfate, 1% MEM nonessential amino acids) were transfected with 100 μl of transfection mix. This mix was maintained for 20 min at room temperature before transfection (800 ng of pSICO‐Flpo plasmid (Addgene plasmid 24969 http://n2t.net/addgene:24969 (Young & Jacks, 2010)), or 800 ng of pFsp1‐Flpo plasmid (Institut Clinique de la Souris; C4632), or 800 ng of pCMV:GFP(FRT)lacZ plasmid (Addgene 31,124; Gerhart Ryffel), 4 μl of Lipofectamine® 2000 Transfection Reagent (Invitrogen), Opti‐MEM medium (Thermofisher) to 100 μl). Medium was changed 6 hr after transfection and replaced by a complete medium (Dulbecco's Modified Eagle's Medium, supplemented with 0.03% l‐glutamine and containing 10% fetal bovine serum, a mix of 100 U ml^−1^ penicillin and 100 μg ml^−1^ streptomycin sulfate, 1% MEM nonessential amino acids). Forty‐eight hours after transfection, wells were rinsed with PBS.

### 3.3 Molecular biology

#### 3.3.1 Genomic and recombination PCR

For gDNA extraction from transfected HaCaT cells: cells where lysed and gDNA extracted using QIAmp DNA Mini Kit (Qiagen). For gDNA extraction from skin for ^FSF^hPLAP recombination, a skin sample was dilacerated and lysed using collagenase D (4 mg/ml) and Dispase II 4 mg/ml for 1 hr at 37°C. The Lysis mix was spun 5 min at 1,500 rpm and the pellet washed with PBS1X. The pellet was suspended in 50 μl lysis buffer (25 mM NaOH, 0.2 mM EDTA) and lysed 30 min at 95°C. Fifty micro liter of neutralization buffer (40 mM Tris–HCl) was then added. DNA extraction and PCR were performed as previously described (Vincent et al., 2010) using Taq DNA polymerase (Life Technologies) and primers cited in Table 1.

**Table 1:** List of primers

	Primer name/alternate name	Primer sequences
Excised and nonexcised *pCMV:GFP(FRT)lacZ*	FWD/p1	CGACACTGCAGAGACCTACT
	REV/p2	GCCTCTTCGCTATTACGCCA
*Fsp1‐Flpo* transgene	FWD/p3	CTGGCCACATTCATCAACTGCGG
	REV/p4	CTTCTTCAGGGCCTTGTTGTAGCTG
*Flpo* mRNA	FWD/p3	CTGGCCACATTCATCAACTGCGG
	REV/p4	CTTCTTCAGGGCCTTGTTGTAGCTG
*Unrecombined* ^*FSF*^ *hPLAP* transgene	FWD/p5	CAGTAGTCCAGGGTTTCCTTGATG
	REV/p6	ACTTCCATTTGTCACGTCCTGCAC
Recombined ^*FSF*^ *hPLAP* transgene	FWD/p5	CAGTAGTCCAGGGTTTCCTTGATG
	REV/p7	CCCAGGAAGATGATGAGGTTCTTG
^*FSF*^ *hPLAP* mRNA	FWD/p8	TCAGTGCGGTTCCACACATA
	REV/p9	ACGCAGCTCATCTCCAACAT
*PDGFRα* mRNA	FWD	TTCAACGGAACCTTCAGCGT
	REV	ACGATCGTTTCTCCTGCCTT
*GAPDH* mRNA	FWD	CCCAGCAAGGACACTGAGCAAGAG
	REV	CTAGGCCCCTCCTGTTATTATGGGG
*Fsp1 mRNA*	FWD	TCTTGGTCTGGTCTCAACGG
	REV	TGTCACCCTCTTTGCCTGAG

#### 3.3.2 RNA extraction

RNA extraction from sorted skin cells: cells from cell sorting where lysed and RNA extracted using the RNeasy Micro Kit (Qiagen). For RNA extraction from transfected cells: total RNA was extracted and purified from cells using lysis buffer from the RNeasy Mini Kit (Qiagen). For RNA extraction from organs: total RNAs were extracted from tissue using ultraturax to mix them in a home‐made RNA extraction solution (Guanidine thiocyanate 5 M [Sigma G6639], Citrate de sodium 0.5 M pH 7.0, Lauryl sarcosine 10%, βmercaptoethanol 1%). Purification was performed using the RNeasy Mini Kit (Qiagen).

#### 3.3.3 Reverse‐transcription PCR

First‐strand cDNAs were prepared using 250 ng of RNA and SuperScript II Reverse Transcriptase in the presence of random primers (Invitrogen) (Vincent et al., 2009). Semi‐quantitative PCR on cDNA was performed as previously described (Pommier et al., 2012) and using an Applied Biosystems StepOnePlus Real‐Time PCR System with MESA GREEN qPCR MasterMix Plus (Eurogentec) or a Roche LightCycler 480 Real time PCR system with Fast SYBR™ Green Master Mix (Life Technologies). All real‐time values were averaged and compared using the threshold cycle (C_T_) method, in which the amount of target RNA (2^−ΔΔCT^) was normalized against the endogenous expression of *GAPDH* (glyceraldehyde‐3‐phosphate dehydrogenase) (ΔC_T_). The amount of target mRNA in control cells or tissues was arbitrarily set at 1.0. Primers used for PCR are listed in Table 1.

#### 3.3.4 Western blotting

Total protein extracts (50 μg) were prepared using RIPA lysis buffer (50 mM Tris, pH 7.5, 150 mM NaCl, 1% Nonidet P‐40, 0.5% sodium deoxycholate, 0.1% SDS and commercial protease and phosphatase inhibitor cocktail tablets [Roche]) and were subjected to SDS‐PAGE. The separated proteins were transferred onto PVDF membranes (Millipore) by electroblotting. Western blots were visualized using the ECL Western detection system (GE Healthcare). Images were captured using a ChemiDoc Biorad MP. We used human placental alkaline phosphatase primary antibody, rabbit monoclonal 1:500 (Abcam ab16695 [SP15]), GAPDH, mouse monoclonal primary antibody, 1:1,000 (Abcam ab8245) and the secondary antibodies were Rabbit IgG (H + L), HRP‐conjugated, 1:5,000 (Ozyme GtxRb‐003‐DHRPX), and anti‐Mouse IgG, HRP‐conjugated, rabbit polyclonal 1:20,000 (Dako P0260).

### 3.4 Histology

For immunofluorescent (IF) staining, OCT‐frozen tissues were sliced into 10 μm‐thick sections. Slides were dried 30 min at room temperature, postfixed in 4% paraformaldehyde in PBS 1X for 10 min at 4°C, then washed in cold PBS 1X, permeabilized with 0.5% Triton X‐100 for 5 min, and blocked with PBS‐4% BSA for 1 h. Labeling was performed by incubating slides overnight with rabbit polyclonal primary antibodies against FSP1/S100A4 (1:200 Abcam ab41532). After three PBS 1X washes, slides were incubated with the specific secondary antibodies Alexa647‐conjugated goat anti‐rabbit antibody (1:1,000 Life Technologies A‐21245 [GAR647]) and DAPI (1:1,000 Sigma d9542). All antibodies were diluted in DakoReal Antibody Diluent. Slides were washed three times with PBS 1X, Samples were mounted on microscope slides with DAKO Fluorescence Mounting Medium (Agilent S3023) or Fluoromount Aqueous mounting medium (Sigma F4680). Images were acquired with a Zeiss Imager M2 AX10 and processed using Zeiss Zen.

Alkaline phosphatase (AP) staining was performed as follows: 10 μm‐thick sections of frozen tissues were cut using the Cryostat HM550 (MICROM). Cryosections were dried 30 min at room temperature, postfixed in 4% paraformaldehyde in PBS 1X for 10 min at 4°C, then washed in cold PBS 1X. Sections were incubated at 70°C in preheated PBS for 90 min, then incubated in AP detection buffer (100 mM NaCl, 100 mM Tris–HCl pH 9.5, 50 mM MgCl2) for 60 min. Sections were subsequently placed in AP staining solution (AP detection buffer with 0.8 mg/ml nitroblue tetrazolium (NBT), 0.1 mg/ml 5‐bromo‐4‐chloro‐3‐indolyl phosphate dimethylformamide (BCIP)) at room temperature for 2–6 hr. Once color development was complete, sections were washed in PBS 1X.

### 3.5 Statistical analysis

All graphs display one representative experiment performed three times. Prism 7.0 (Graphpad) was used for statistics and to create graphs. The error bars represent the standard deviation (SD) from technical duplicates.

## CONFLICT OF INTEREST

The authors declare no conflict of interest.

## AUTHOR CONTRIBUTIONS

Victoire Cardot‐Ruffino, Stéphanie Sentis, and Laurent Bartholin designed the experiments. Victoire Cardot‐Ruffino, Véronique Chauvet, Cassandre Caligaris, Sylvie Martel, and Sophie Aires performed the experiments. All authors analyzed the experimental results. Laurent Bartholin, Victoire Cardot‐Ruffino, and Stéphanie Sentis wrote the article. All authors were involved in critical reading of the article prior to submission.

## Supporting information


**Appendix** S1: Supporting Information
