# Supplementary material for: Generation of an Fsp1 (fibroblast‐specific protein 1)‐Flpo transgenic mouse strain
Source: Genesis. 2020 Mar 19;58(5):e23359. doi: 10.1002/dvg.23359 (PMC7317532; doi:10.1002/dvg.23359)
Supplement: Supplementary file 1 — Appendix S1: Supporting Information [file DVG-58-e23359-s001.docx]

**Sequencing of [*Fsp1-FLPo*] transgene plasmid**

**IM3516/U147 ICS**

tgcctgtagcaatggcaacaacgttgcgcaaactattaactggcgaactacttactctagcttcccggcaacaattaatagactggatggaggcggataaagttgcaggaccacttctgcgctcggcccttccggctggctggtttattgctgataaatctggagccggtgagcgtgggtctcgcggtatcattgcagcactggggccagatggtaagccctcccgtatcgtagttatctacacgacggggagtcaggcaactatggatgaacgaaatagacagatcgctgagataggtgcctcactgattaagcattggtaactgtcagaccaagtttactcatatatactttagattgatttaaaacttcatttttaatttaaaaggatctaggtgaagatcctttttgataatctcatgaccaaaatcccttaacgtgagttttcgttccactgagcgtcagaccccgtagaaaagatcaaaggatcttcttgagatcctttttttctgcgcgtaatctgctgcttgcaaacaaaaaaaccaccgctaccagcggtggtttgtttgccggatcaagagctaccaactctttttccgaaggtaactggcttcagcagagcgcagataccaaatactgtccttctagtgtagccgtagttaggccaccacttcaagaactctgtagcaccgcctacatacctcgctctgctaatcctgttaccagtggctgctgccagtggcgataagtcgtgtcttaccgggttggactcaagacgatagttaccggataaggcgcagcggtcgggctgaacggggggttcgtgcacacagcccagcttggagcgaacgacctacaccgaactgagatacctacagcgtgagctatgagaaagcgccacgcttcccgaagggagaaaggcggacaggtatccggtaagcggcagggtcggaacaggagagcgcacgagggagcttccagggggaaacgcctggtatctttatagtcctgtcgggtttcgccacctctgacttgagcgtcgatttttgtgatgctcgtcaggggggcggagcctatggaaaaacgccagcaacgcggcctttttacggttcctggccttttgctggccttttgctcacatgttctttcctgcgttatcccctgattctgtggataaccgtattaccgcctttgagtgagctgataccgctcgccgcagccgaacgaccgagcgcagcgagtcagtgagcgaggaagcggaagagcgcccaatacgcaaaccgcctctccccgcgcgttggccgattcattaatgcagctggcacgacaggtttcccgactggaaagcgggcagtgagcgcaacgcaattaatgtgagttagctcactcattaggcaccccaggctttacactttatgcttccggctcgtatgttgtgtggaattgtgagcggataacaatttcacacaggaaacagctatgaccatgattacgccaagcgcgcaattaaccctcactaaagggaacaaaagctggagctgcggccgcagatctgtcgagcctgcaggggccgattgggccgatatcgctagcttaattaa

***Fsp1 promoter*/Exon 1/Intron 1/Partial Exon 2**

*ccaggtgttgtgatgggaaacgatgggagggagtcagagtcagagggaggctcccagttggggtgaaggtggtagatattctgctcctgaggaatttatggtgatgtcagggaactgaaacttctgtctagaaaaatcacaacaggcacagcggggaggtgaattgattccttgcgagtcatacccttgttttgtgacagtgaggatgacccggcccctggagcaggcagtagctgccatcgtgtgcaccttccaggagtatgcagggcgctgtggggataaatacaagatctgccagtcggagctcaaggagttgttgcagaaggagctgcccacctggacgccggtgagcacctcatatctctccccacttggactctgcaaatcgtggcctagggccaagcagcagcatagggtagcacatgagctgagacacagggctgggaagggggactgaaatgggtgtcttatcaggtgggtaggatgaggttggccccatgagggttttggtttggaatggggcagcctaaccatcggaggggagtggtgtgggaagctactttggctccttattctggtttcctgaccctgccttctcatcccttccacagagtgagttccgggagtgtgactacaataaattcatgagtgttctggataccaacaaagactgcgaagtggactttggggagtacgtgcgctcacttgccagcctctgtctctactgccacgagtacttcaaagagtgcccccctgagcctccttgcccccagtagcctctgatccaaaggtgtacgctatcccagaagggcagggtctgctccagtcctccatctttgtccttgaggtggtcctgggtgtgtagccacacccttcccactctctctgtggtacccctttcaatctagacttgccaagttcttgatgtgctaaccccacccagctacccatgagctttcgaggctttcctagggatgtctagcttgtgaggggtgggacagtagccagcctttgccgcttctcttcttggaagggaagaacatctctgctcagccatgtgcacacaactgggaccgctgtggcaggggcctcctcaactcccaataaagaaatgtcttcttggcttacttttgtttttttctgatgggacacactgggccttgggaccgagtccttgttcctttatgctccttactactggaggtaggaggcttaccatggaaggcatggacccccaaagcggtgtcaggccctgtagaaatgcacacatttcaggagggtaggggtaacacgtgtcctatcagatgagactggagggtctctgtctctctctgtcccctgtcttgagatagaagtccttatcttggactttcaaggaggacaaggggctccttgggaggtacttctgaccagatgctgcaaggagagtatggttgtgggagcccaaagccaaacctccatctaaccttcactcaatccccgaatttgtaccctatccttagagattaatcctgactcccccttttacctatttcctctttaactctcttcttcaagctgaacattcaaccccgaatgctcctgtcattcctcaatatccttactccagcttccatccattcgaaaacctccaggccacactgccaccctaactccatcatggcctcctaggtatagctcctacttcatacctggggtggtcccaaggtccctcctgacttgctagcctctatcctgggtcttcctgattgtgacaagaagctgtttaggctggagggaagtgctgacattgtcccactggctggggtcacctccttcgttcctgggccacatatttccagggcagctccttatcccttgcccataacatctccatctcctttcctgtggcccacacctcatgtccaggttgcccgttctcaaagcttcctaaacttctggctgagctgtggctgcttggtggtgtccaccccatccaagtctctgccgtgcccactggagctcactcactacttgattgtgcctgctggggagggagcaggaagcctggttcccagactgggctggtcgagggtgctatgacatttactacatcaaccaacagcaagagcacagtatccatgttcccccatcctctgcatgggcagggcctggcagggtataaataggtcagattgttgggctctcccca***aacctctctattcagcacttcctctctcttggtctggt**gagttgtgttggtctgatagcactgctagcggcattagaggctgaggctagggtagaagaaaggggggctgctgggggaacagatgtctttaataaatccagatgagagattctgatgtggaggttcatgtatgtgtgtgtgtgtgtgtgtttttcacgagaatgaaaaccaaaaaaaaaaaaaaaaaaaagtgtataaatggctacatctgagctcccggaggttttgagatactgaggctggcttgcatgttgctatagtgtatattggtggtgcttgggagtcactgtcatgcataggatgctgactcgtgttgctgggtaatacaagacagtgtgtggacactcgggtacaggaagcaaagcgaaggcatcagtaggcctttttgttttacagtatttaaattacagtttttatttgtgtgtatgagcgtatgggttgggctggagcaaatgccaaggcgacattgtgggagccaaaggacaatttgtgtgggagtcaatctgttccttctagcatgtgggctgtggggatcaaactcaggccctggagcttggtggcaagcacctctacccactgagctatctctccagcaccctcctgcaggcattcgtgtttgtagtgtgtcttatttttaatagccctatgaacatatagcacctaggccaagaaagcctggcttccccaccctctcctcttgcatccctacctctgccacttcatcttactcctattaggcagctggggtttttccacttttttttgtctgcctctgggcaggcagccagcagccgcgcccaacgctgggagggagaagaatgggccagggcctggtgcttgtggttgagctgtgggagtgagtaagctgatggaaaactgctgttgttgaggccaggactgagaggcacagaaaggtgctggcatggatctccagagtttgaggggtaggctttgcaggtttcagagcccagagcacatgtgaccttcttgccatcaatgggtcccattcctctgatctccccagggggtgaggtccatctcttagagagttggctgggatagagcacttaaaatggggacagaatgagtgtgattt**gggtcatgctcagcaacacatatccagttctcaacacactgttggcgtgggttggagaatgttacttttgtgtctcctgcccttaggtctcaacggttacc**

**FLPo/SV40 polyA**

gaattcgccgccaccatgagccagttcgacatcctgtgcaagaccccccccaaggtgctggtgcggcagttcgtggagagattcgagaggcccagcggcgagaagatcgccagctgtgccgccgagctgacctacctgtgctggatgatcacccacaacggcaccgccatcaagagggccaccttcatgagctacaacaccatcatcagcaacagcctgagcttcgacatcgtgaacaagagcctgcagttcaagtacaagacccagaaggccaccatcctggaggccagcctgaagaagctgatccccgcctgggagttcaccatcatcccttacaacggccagaagcaccagagcgacatcaccgacatcgtgtccagcctgcagctgcagttcgagagcagcgaggaggccgacaagggcaacagccacagcaagaagatgctgaaggccctgctgtccgagggcgagagcatctgggagatcaccgagaagatcctgaacagcttcgagtacaccagcaggttcaccaagaccaagaccctgtaccagttcctgttcctggccacattcatcaactgcggcaggttcagcgacatcaagaacgtggaccccaagagcttcaagctggtgcagaacaagtacctgggcgtgatcattcagtgcctggtgaccgagaccaagacaagcgtgtccaggcacatctactttttcagcgccagaggcaggatcgaccccctggtgtacctggacgagttcctgaggaacagcgagcccgtgctgaagagagtgaacaggaccggcaacagcagcagcaacaagcaggagtaccagctgctgaaggacaacctggtgcgcagctacaacaaggccctgaagaagaacgccccctaccccatcttcgctatcaagaacggccctaagagccacatcggcaggcacctgatgaccagctttctgagcatgaagggcctgaccgagctgacaaacgtggtgggcaactggagcgacaagagggcctccgccgtggccaggaccacctacacccaccagatcaccgccatccccgaccactacttcgccctggtgtccaggtactacgcctacgaccccatcagcaaggagatgatcgccctgaaggacgagaccaaccccatcgaggagtggcagcacatcgagcagctgaagggcagcgccgagggcagcatcagataccccgcctggaacggcatcatcagccaggaggtgctggactacctgagcagctacatcaacaggcggatctgaggatccagatcttattaaagcagaacttgtttattgcagcttataatggttacaaataaagcaatagcatcacaaatttcacaaataaagcatttttttcactgcattctagttgtggtttgtccaaactcatcaatgtatcttatcatgtctggtcgaggcggccgcgtacccaattcgccctatagtgagtcgtattacgcgcgctcactggccgtcgttttacaacgtcgtgactgggaaaaccctggcgttacccaacttaatcgccttgcagcacatccccctttcgccagctggcgtaatagcgaagaggcccgcaccgatcgcccttcccaacagttgcgcagcctgaatggcgaatggaaattgtaagcgttaatattttgttaaaattcgcgttaaatttttgttaaatcagctcattttttaaccaataggccgaaatcggcaaaatcccttataaatcaaaagaatagaccgagatagggttgagtgttgttccagtttggaacaagagtccactattaaagaacgtggactccaacgtcaaagggcgaaaaaccgtctatcagggcgatggcccactacgtgaaccatcaccctaatcaagttttttggggtcgaggtgccgtaaagcactaaatcggaaccctaaagggagcccccgatttagagcttgacggggaaagccggcgaacgtggcgagaaaggaagggaagaaagcgaaaggagcgggcgctagggcgctggcaagtgtagcggtcacgctgcgcgtaaccaccacacccgccgcgcttaatgcgccgctacagggcgcgtcaggtggcacttttcggggaaatgtgcgcggaacccctatttgtttatttttctaaatacattcaaatatgtatccgctcatgagacaataaccctgataaatgcttcaataatattgaaaaaggaagagtatgagtattcaacatttccgtgtcgcccttattcccttttttgcggcattttgccttcctgtttttgctcacccagaaacgctggtgaaagtaaaagatgctgaagatcagttgggtgcacgagtgggttacatcgaactggatctcaacagcggtaagatccttgagagttttcgccccgaagaacgttttccaatgatgagcacttttaaagttctgctatgtggcgcggtattatcccgtattgacgccgggcaagagcaactcggtcgccgcatacactattctcagaatgacttggttgagtactcaccagtcacagaaaagcatcttacggatggcatgacagtaagagaattatgcagtgctgccataaccatgagtgataacactgcggccaacttacttctgacaacgatcggaggaccgaaggagctaaccgcttttttgcacaacatgggggatcatgtaactcgccttgatcgttgggaaccggagctgaatgaagccataccaaacgacgagcgtgacaccacg

## Fig S1: Verified sequence of the Fsp1-Flpo transgene cloned inside the recombination vector.

Result of ICS sequencing. Fsp-1 (Fibroblast Specific Promoter 1)- Flpo (Flipo recombinase) sequence - SV40 (Simian Virus 40) polyA (polyadenylation signal). Primer sequences used for PCR and RT-PCR are underlined.
